# Towards Effective *Helicobacter pylori* Eradication: Emerging Therapies in the Wake of Antibiotic Resistance

**DOI:** 10.3390/ijms26136064

**Published:** 2025-06-24

**Authors:** Barathan Muttiah, Wathiqah Wahid, Asif Sukri, Alfizah Hanafiah

**Affiliations:** 1 Department of Medical Microbiology and Immunology, Faculty of Medicine, Universiti Kebangsaan Malaysia, Cheras, Kuala Lumpur 56000, Malaysia; 2 Department of Medical Parasitology and Entomology, Faculty of Medicine, Universiti Kebangsaan Malaysia, Cheras, Kuala Lumpur 56000, Malaysia; wathiqah.wahid@hctm.ukm.edu.my; 3 Department of Biological Sciences and Biotechnology, Faculty of Science and Technology, Universiti Kebangsaan Malaysia, Bangi 43600, Malaysia; mohdasifsukri@ukm.edu.my; 4 GUT Research Group, Universiti Kebangsaan Malaysia, Cheras, Kuala Lumpur 56000, Malaysia

**Keywords:** *Helicobacter pylori*, antibiotic resistance, gut microbiota, extracellular vesicles, probiotics, alternative therapies

## Abstract

*Helicobacter pylori* (*H. pylori*) infection is a leading cause of gastritis, peptic ulcers, and gastric cancer, affecting more than half of the global population. Its persistence in the acidic gastric environment and its ability to evade host immunity present major treatment challenges. Although antibiotics remain the standard therapy, rising antimicrobial resistance has reduced treatment efficacy, prompting the search for alternative and adjunct approaches. Emerging therapies include probiotics, antimicrobial peptides (AMPs), and plant-derived compounds, which target *H. pylori* through membrane disruption, immunomodulation, or direct antimicrobial activity. Novel drug delivery systems and microbiota-sparing interventions are also being investigated. Additionally, vaccine development offers a promising strategy for long-term protection, though challenges related to antigenic variability and host-specific responses remain. Despite these advances, treatment variability and the limited clinical validation of alternatives hinder progress. A multifaceted approach integrating microbiome research, host–pathogen interactions, and new therapeutic agents is essential for future success.

## 1. *Helicobacter pylori* Infection

*Helicobacter pylori* (*H. pylori*) is a common, chronic bacterial infection that significantly contributes to various gastrointestinal disorders. The infection can range from asymptomatic gastritis to severe conditions such as peptic ulcers, gastric mucosa-associated lymphoid tissue (MALT) lymphoma, and gastric cancer [1]. The transmission routes are predominantly via direct contact, oral–oral routes, and contaminated water or food sources. One study mentioned that approximately 50% of the world’s population is estimated to be infected with *H. pylori* [2]. Specifically, *H. pylori* infection is significantly more prevalent, often reaching 80–90%, in developing countries like Africa, South Asia, and Latin America. Factors such as poor sanitation, contaminated water, crowded living conditions, co-infections (soil-transmitted helminths), and limited healthcare access contribute to this high prevalence [3,4]. In contrast, developed countries, including those in Western Europe and North America, exhibit much lower prevalence rates, ranging from 20% to 40% due to improved sanitation, better hygiene practices, and greater healthcare access [5]. Additionally, a decreasing trend in *H. pylori* prevalence is observed in these regions due to improved hygiene, healthcare access, and awareness [6].

In general, *H. pylori* infection is acquired during childhood, with infection rates increasing with age. Studies have shown that the highest incidence of seroconversion occurs in early childhood [7]. As children grow older, the rate of new infections decreases, but the overall prevalence increases with age due to the cumulative effect of acquiring the infection over time [1]. Conversely, higher prevalence has been noted in populations with a family history of gastric diseases, such as gastric cancer, peptic ulcer disease, and relapses in previously treated individuals. This is likely due to genetic factors that may make some individuals more susceptible to infection and its complications [8]. The infection is also linked to other gastrointestinal issues like duodenal ulcers and vitamin B12 deficiency [6]. Diagnosis involves both invasive and non-invasive methods, including stool antigen tests, PCR stool tests, urea breath tests, and upper gastrointestinal procedures such as endoscopy or X-rays [9]. Currently, urease testing and histological analysis are widely regarded as the gold standard in diagnosing *H. pylori* infection in many clinical situations. However, no single method can be universally defined as the gold standard for diagnosis. Non-invasive tests such as the urea breath test and stool antigen test are highly recommended and commonly used [10]. Further evidence is needed before any diagnostic method can be established as the definitive gold standard for diagnosing *H. pylori* infection across different clinical scenarios. The infection is typically treated with a combination of antibiotics and acid suppressants to eradicate the bacteria and promote stomach healing [11].

## 2. Current Therapeutic Approaches for *H. pylori* Infection

**Figure 1:**
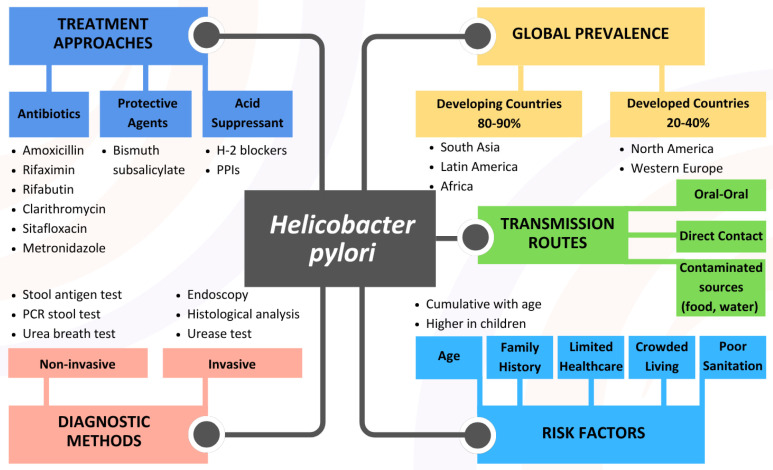
The overview of *H. pylori* infection.

Common antibiotics used include amoxicillin (AMX), clarithromycin (CLR), metronidazole (MTZ), rifabutin, rifaximin, and sitafloxacin, with the latter three being considered for third-line treatment [12]. Proton pump inhibitors (PPIs), such as omeprazole and esomeprazole, help reduce stomach acid, while bismuth subsalicylate (Pep-to-Bismol) protects ulcers from acid. Histamine (H-2) blockers, such as cimetidine, block acid production [13]. On the other hand, potassium-competitive acid blockers (P-CABs), such as Vonoprazan, play a crucial role in contemporary *H. pylori* eradication therapies [12]. When combined with antibiotics such as AMX and CLR, it often outperforms traditional PPIs, contributing to higher eradication rates and improved treatment efficacy. Among all these, rifabutin-based and CLR-based triple therapies are the most common [14]. However, there is no treatment that guarantees a 100% cure rate, and antibiotic resistance must be considered when selecting a therapeutic approach. Figure 1 illustrates a comprehensive overview of *H. pylori* infection and global prevalence, showing high infection rates in developing countries (80–90% in Africa, South Asia, and Latin America) and lower rates in developed countries (20–40% in Western Europe and North America); transmission routes, detailing direct contact, oral–oral transmission, and contamination through water and food; risk factors, including poor sanitation, overcrowded living conditions, limited healthcare access, family history, and age-related factors (higher rates in children, cumulative increase with age); diagnostic methods, distinguishing between non-invasive approaches (stool antigen test, PCR stool test, urea breath test) and invasive procedures (endoscopy, urease testing, histological analysis); and treatment approaches, categorizing options into antibiotics (amoxicillin, clarithromycin, metronidazole, rifabutin, rifaximin, sitafloxacin), acid suppressants (PPIs and H-2 blockers), and protective agents (bismuth subsalicylate), providing a complete picture of therapeutic strategies.

## 3. Antibiotic Resistance in *H. pylori*

The prevalence of antibiotic resistance in *H. pylori* varies significantly worldwide, often exceeding 95% in certain regions, particularly in low- and middle-income countries where the misuse and overuse of antibiotics are prevalent [15]. This widespread resistance compromises the efficacy of standard eradication therapies and heightens the risk of treatment failure. However, the emergence of resistance to commonly used antibiotics, such as MTZ, CLR, and levofloxacin (LVX), has drastically reduced treatment success rates worldwide. AMX is widely used as a first-line treatment for *H. pylori* eradication owing to its affordability and safety, and resistance remains low (0–10%) in most countries, with low resistance observed in Asia (~3%), China (2.8%), Europe (~0.4%), and Algeria (0%), but high rates reported in Vietnam (25.7%) and Africa (up to 100%) [16]. Resistance arises through three primary mechanisms, which are mutations in penicillin-binding proteins (PBPs), particularly PBP1A, which reduce AMX binding affinity via amino acid substitutions (S402G, T555S); followed by β-Lactamase production such as the *blaTEM-1* gene, which degrades AMX and may spread via horizontal gene transfer; and lastly, porin and efflux pump modifications, where mutations in proteins like helicobacter outer membrane protein B (HopB), helicobacter efflux protein A (HefA), and outer membrane protein (omp25) decrease AMX permeability and intracellular accumulation. Long-term low-dose exposure further alters porin expression, complicating treatment outcomes [17,18].

MTZ, a nitroimidazole antibiotic, shows mixed trends globally, particularly in East and Southeast Asia, with rates reaching 87.8% in China, 94.6% in Bangladesh, and 81.6% in India. In Europe, resistance averages 32%, peaking in France (58.6%) and falling to 10.2% in Austria. Oceania reports ~50% resistance, while the Americas average 23% but decreases in Chile and Spain [19]. Resistance is particularly prevalent in developing countries compared to Western nations, possibly due to the widespread and frequent use of MTZ for other infections, which increases selective pressure on *H. pylori* [20]. Patients who have previously undergone treatment for *H. pylori* are more likely to develop resistance, as are those diagnosed with nonulcerated duodenal ulcers (NUD). MTZ resistance primarily results from genetic mutations in the *rdxA, frxA*, or *fdxB* genes, which result in a reduction of nitroreductase activity, leading to the decreased activation of the drug and rendering it ineffective [21]. The high expression of efflux pump genes like *hp1165* and *hefA* also contributes to resistance and is associated with multidrug resistance [22].

CLR resistance has risen in many regions, particularly in China and treated patients in France and Taiwan [23]; also, some significant regional variations were observed, including about 18% in Europe, 33% in the Eastern Mediterranean, 34% in the Western Pacific, less than 10% in the Americas and Southeast Asia, and less than 29.2% in Africa, though specific countries like Congo reported lower rates (1.7%) [24]. Resistance primarily arises from mutations in the *23S rRNA* gene (e.g., A2142G, A2143G), which hinder CLR binding, along with synergistic mutations in genes like *rpl22* and *infB* [25]. Efflux pump systems, particularly the RND family, actively expel CLR, reducing its efficacy, with inhibitors like PaβN and CCCP showing the potential to reverse this effect. OMP alterations, such as changes in HopT and OMP31 expression, further contribute to resistance by decreasing membrane permeability [26]. These mechanisms collectively undermine CLR’s effectiveness, posing challenges for *H. pylori* treatment.

Tetracycline (TET) resistance remains low globally, except for notable increases in Iran and China, ranging from 0.6% to 8%, and in Europe (below 1%). The United States and Australia also report low resistance, making it a preferred option in quadruple therapy, achieving over 90% eradication success even in areas with high resistance to other antibiotics like CLR and MTZ [27]. TET’s mechanism of action involves inhibiting protein synthesis by binding bacterial ribosomes, but resistance occurs mainly through mutations in the *16S rRNA* gene that prevent TET binding [28]. High-level resistance results from triple mutations at the TET binding site, while lower resistance is linked to double or single mutations. Additionally, TET resistance can be enhanced by proton motive force-dependent efflux pump systems, with the *Hp1165* gene playing a key role in some strains [19,29]. However, other efflux pump determinants may also contribute to resistance, suggesting a complex mechanism of TET resistance in *H. pylori*.

LVX, a fluoroquinolone antibiotic resistance, has escalated in Europe, Asia, and the Western Pacific, exceeding 30% in countries like China (30.29%), India (54.9%), the U.S. (37.6%), and Iran (18%), while lower rates are observed in Pakistan, Thailand, and Brazil [23]. Due to high resistance, LVX is mainly used in remedial treatments after initial failure or based on drug susceptibility tests. Resistance arises primarily from mutations in the quinolone resistance determination region (QRDR) of the *gyrA* gene, particularly at amino acids 87 and 91 (N87K, D91G), and sometimes in the *gyrB* gene [30]. These mutations prevent LVX from binding to DNA gyrase, rendering the antibiotic ineffective [31]. Other mutations in *gyrA* and *gyrB* also contribute to LVX resistance, though their impact requires further investigation [32].

Multidrug resistance (MDR) is rising, with double and triple resistance patterns becoming more prevalent, especially in regions like Chile and Peru [33]. Several studies have highlighted significant antibiotic resistance in *H. pylori* strains across various regions. In Isfahan, Iran, resistance rates for CLR, MTZ, and AMX were 15.3%, 55.1%, and 6.4%, respectively, with one multidrug-resistant isolate identified [34]. A study in Myanmar revealed high MDR, particularly to MTZ and LVX, with 84.2% showing dual-drug resistance [35]. In Saudi Arabia, 8.8% of isolates were MDR, with high resistance to MTZ (48.2%) and CLR (27.7%) [36]. Italy demonstrated an increase in resistance over time, particularly for CLR (35.9%) and MTZ (40.2%) [37]. Meanwhile, in Malaysia, a study reported high resistance to MTZ (82.4%) and CLR (72.5%), with 82.4% of isolates exhibiting multidrug resistance [38]. Resistance rates were 65.2% for MTZ and 34.8% for LVX, with no resistance observed for AMX in a study conducted in Shanghai [39]. These trends highlight the urgent need for tailored treatment strategies and enhanced antibiotic stewardship to combat *H. pylori* resistance. The challenges associated with broad-spectrum antibiotic therapy are particularly pronounced in pediatric patients, where the loss of gut flora during extended treatments poses significant health risks. Moreover, the increasing failure of eradication strategies and the desire to prevent gastric cancer have spurred interest in more targeted therapeutic approaches, such as local drug delivery methods. Table 1 provides antibiotic resistance patterns and mechanisms against *H. pylori*.

## 4. Biofilms of *H. pylori*

**Figure 2:**
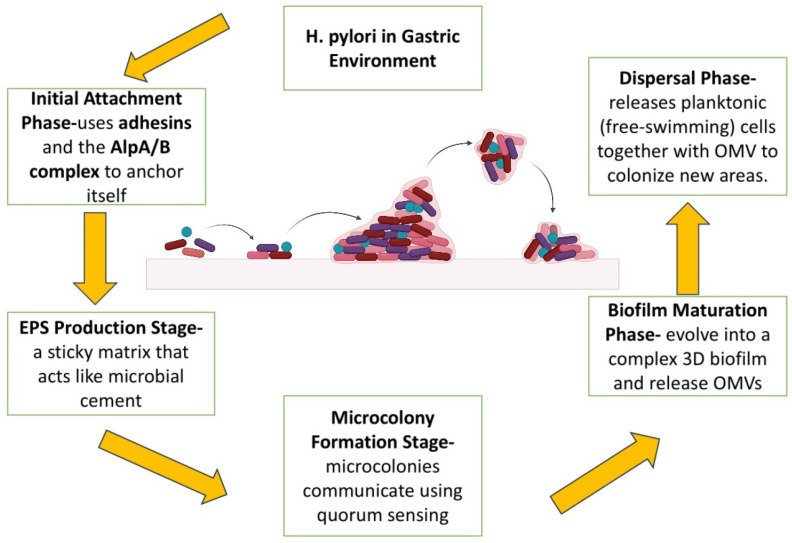
*H. pylori* biofilm formation. The sequential stages of *H. pylori* biofilm formation within the gastric environment, beginning with initial attachment where the bacterium uses adhesins such as BabA, SabA, and the AlpA/B complex to anchor itself to biotic or abiotic surfaces, aided by flagellar motility and chemotaxis. This is followed by the EPS production stage, where *H. pylori* secrete extracellular polymeric substances (EPS) including eDNA, proteins, and polysaccharides, forming a sticky matrix that acts like microbial cement. In the microcolony formation stage, bacterial cells aggregate into clusters and communicate via quorum sensing through autoinducer-2 (AI-2) and the *LuxS* gene product. These microcolonies mature into complex 3D biofilm structures in the biofilm maturation phase, characterized by increased antibiotic resistance and the release of outer membrane vesicles (OMVs) carrying virulence factors such as CagA, VacA, LPS, and outer membrane proteins (OMPs). Finally, during the dispersal phase, nutrient depletion or stress triggers the release of single, planktonic cells along with OMVs, enabling the colonization of new niches and perpetuating the infection cycle.

*H. pylori* biofilms are now recognized as one of the most important causes of antibiotic resistance and relapse infection [40]. Biofilm growth is a survival strategy that enables *H. pylori* to colonize and persist in the hostile gastric environment [41]. It also acts as a shield against host immunity and acid stress and eventually inhibits drug penetration, enhances metabolic dormancy, and enhances phenotypic heterogeneity that eventually decreases therapeutic effectiveness [42]. The structures are also involved in transmission and reinfection and have been observed not only on gastric mucosa but also abiotically on water systems, serving as reservoirs in the environment [43,44]. Mature biofilm dispersal allows for intra-host transmission as well as inter-host transmission [45,46]. *H. pylori* biofilm development is a multi-step process initiated by bacterial adhesion to surfaces, stimulated by outer membrane adhesins such as BabA, SabA, and AlpA/B [47]. Motility and chemotaxis via chemoreceptors such as TlpA–D also favor niche localization [48]. The matrix of the biofilm consists of exopolysaccharides (EPS), extracellular DNA (eDNA), proteins, and OMVs, along with other substances [49,50]. OMVs, formed through outer membrane blebbing, represent the predominant type of bacterial extracellular vesicles (bEVs) produced by Gram-negative bacteria such as *H. pylori* [51]. OMVs are lipidated with lipopolysaccharides (LPS), outer membrane proteins (OMPs), and virulence factors such as CagA, VacA, and urease, playing a multifunctional role in biofilm integrity, bacterial survival, and modulation of the host immune system [52]. EPS and eDNA are involved in matrix cohesion and horizontal gene transfer, enabling adaptability and resistance [53,54,55]. Quorum sensing through the AI-2 signaling system, provided by luxS, controls biofilm architecture and virulence. TlpB, in particular, perceives AI-2 as a chemorepellent and affects dispersal dynamics; luxS mutation enhances biomass and cluster formation, highlighting its regulatory function [56,57]. Biofilms, when mature, form 3D architectures with microcolony heterogeneity, reduced antibiotic permeability, and enhanced expression of efflux pumps [58,59]. Dispersal, generally induced by stress or starvation, is regulated by ECM-degrading enzymes like proteases and DNases, releasing coccoid cells in a resting state with enhanced resistance and persistence potential [60,61,62]. Figure 2 illustrates the complete biofilm formation life cycle from adhesion to dispersal through ECM maturation.

### Factors Influencing Formation of Biofilms

The development of *H. pylori* biofilms is driven by environmental stresses such as gastric acidity, nutrient starvation, and intercellular communication, which together enhance bacterial survival and promote chronic infection [63,64,65,66].

*H. pylori*’s metabolic flexibility and its ability to form biofilms on both biotic and abiotic surfaces, including medical devices, expand its ecological niches and enhance its potential for persistence [67]. The virulence factors VacA and CagA play synergistic roles in promoting biofilm formation and gastric pathogenesis [68,69,70]. CagA, delivered into gastric epithelial cells via the type IV secretion system, disrupts cellular polarity, tight junctions, and cytoskeletal structures. It also controls NF-κB-mediated inflammatory responses, which can induce carcinogenesis [68,69,70,71].

VacA induces vacuolation, disrupts endolysosomal trafficking, and suppresses autophagy, leading to the intracellular accumulation of CagA and increased cytotoxicity [72,73,74]. In combination, these factors augment mucosal damage and are associated with more severe gastroduodenal disease [75]. Notably, sub-therapeutic antibiotic exposure can paradoxically induce biofilm formation by triggering stress responses and enhancing matrix production [76].

Under such stress, *H. pylori* may adopt a viable but non-culturable (VBNC) coccoid form, in which it is metabolically inactive but capable of reactivation. VBNC cells contribute matrix material to biofilms through lysis, releasing DNA and proteins and thereby strengthening biofilm integrity [77,78]. This mutual relationship stabilizes biofilms and promotes immune evasion. VBNC cells are highly antibiotic-resistant and evade detection by standard culture methods, often leading to treatment failures, relapses, and delayed diagnoses [79,80,81,82,83].

Together, *H. pylori* biofilms, facilitated by the coordination of virulence factors, environmental stress responses, and the VBNC state, form a chronic reservoir that is resistant to eradication and contributes to long-term gastric disease [84,85].

## 5. The Influence of Bacterial Extracellular Vesicles During *H. pylori* Infection

bEVs are nanosized, membrane-bound particles secreted by Gram-negative and Gram-positive bacteria. They harbor diverse biomolecular cargo, including lipids, proteins, toxins, nucleic acids, and signaling molecules [86,87]. Initially believed to be unique to Gram-negative bacteria due to their outer membrane, bEVs are now recognized as universal structures across bacterial species, with Gram-positive bacteria using autolysins and endolysins to traverse their thick peptidoglycan walls [88]. These vesicles are central mediators of bacterial physiology, facilitating intercellular communication both among microbial communities and at host–microbe interfaces [89]. In *H. pylori*, OMVs, a subcategory of bEVs, are the major secreted vesicle type and play a critical role in the delivery of virulence, immune evasion, and biofilm formation.

Functionally, bEVs participate in nutrient acquisition, stress responses, biofilm development, and horizontal gene transfer. They also serve as delivery vehicles for virulence factors, toxins, and immunomodulatory molecules, thereby affecting bacterial pathogenicity and the host immune response [90,91]. bEVs also contain pathogen-associated molecular patterns (PAMPs) that interact with host pattern recognition receptors, inducing innate and adaptive immunity, thereby a dual function in both pathogenesis and immunoregulation [92].

In Gram-negative bacteria like *H. pylori*, bEVs are primarily OMVs, which are generated through outward blebbing of the outer membrane. The outer membrane is an asymmetric bilayer composed of phospholipids in the inner leaflet and lipopolysaccharides (LPS) in the outer leaflet, together with OMPs involved in adhesion, nutrient uptake, and environmental sensing [93,94]. OMVs from these membranes include a mixture of bioactive molecules, including LPS, OMPs, phospholipids, peptidoglycan fragments, enzymes, and toxins such as VacA and CagA, which are key players in *H. pylori* pathogenesis [95]. OMV biogenesis is a regulated process of vesicle formation via membrane curvature and pinching that enables the bacterium to release toxic or immunomodulatory effectors in a controlled manner [95].

Recent studies have extended the understanding of vesicle heterogeneity in Gram-negative bacteria. Among them is the outer–inner membrane vesicle (OIMV), containing outer and inner membrane material, likely produced by explosive lysis propelled by phage-encoded endolysins. This vesicle contains abundant cytoplasmic cargo, including DNA, cytosolic proteins, and enzymes [96,97]. One of these new subtypes is the explosive outer membrane vesicle (EOMV), which also derives from cell lysis events, yet differs from OMVs in its disordered, unstructured assembly. EOMVs encapsulate large, random quantities of cellular contents and can be agents of horizontal gene transfer or bactericidal activity towards competing microbes [98,99,100].

Although Gram-positive bacteria lack an outer membrane, they also shed vesicles, which are commonly referred to as cytoplasmic membrane vesicles (CMVs). These vesicles are discharged through processes of “bubbling cell death,” a form of autolysis in which bulging of the cytoplasmic membrane leads to vesicle release, mediated by autolysins that degrade the peptidoglycan layer [101,102,103]. Interestingly, vesicle formation in Gram-positive bacteria is not solely associated with cell death; viable cells can also release CMVs as part of regulated secretion processes involved in immune modulation, biofilm formation, and intercellular communication [104,105].

While the therapeutic promise of bEVs, particularly *H. pylori*-derived OMVs, is becoming more apparent in drug delivery, vaccine engineering, and biomarker identification, there are several challenges that must be surmounted. These include the heterogeneity of vesicle populations, scalability of production and purification, guarantee of safety and specificity, and information on their in vivo kinetics and mode of action [106]. There is a need for the standardization of bEV isolation protocols and for understanding their roles in health and disease through more studies. Nonetheless, bEVs represent a promising avenue for future antimicrobial and immunotherapeutic strategies to *H. pylori* and other persistent infections. Table 2 explains the multifaceted roles of bEVs in the context of *H. pylori* infection, from their composition and function to their potential in diagnostics and treatment.

### Role of OMVs in H. pylori Pathogenesis, Biofilm Formation, and Antibiotic Resistance

*H. pylori* have evolved sophisticated mechanisms to survive the gastric acidic environment, and OMVs play a key role in host–pathogen interactions. OMVs are nanometer-sized (20–300 nm) vesicles that are formed by the outward blebbing of the bacterial outer membrane, and they contain a heterogeneous cargo of virulence factors such as CagA, VacA, urease subunits, γ-glutamyltransferase (GGT), and adhesins such as BabA and SabA that enhance bacterial adhesion to host epithelial cells [107,108]. In addition to presenting virulence factors, OMVs also transport immunomodulatory molecules that can induce inflammation for colonization and also allow immune evasion [109].

OMVs are carriers of PAMPs such as LPS and peptidoglycan that activate host pattern recognition receptors (PRRs) such as TLRs and NOD1 [110]. This triggers inflammatory cascades mediated by NF-κB, caspases, and cytokines such as IL-8, IL-6, IL-1β, and TNF-α, facilitating immune cell recruitment and inflammation [110,111]. Conversely, OMVs allow for immune suppression by activating heme oxygenase-1 (HO-1) via Akt-Nrf2 and mTOR–IKK–NF-κB signaling pathways in dendritic cells and macrophages [112]. Moreover, OMV-associated factors like GGT and VacA induce T cell apoptosis, cell cycle arrest, and disordered proliferation, while small non-coding RNAs and nucleic acids modulate host gene expression, inhibit autophagy, and interfere with antigen presentation by the interaction of, for instance, VacA with CD18 on T cells [113,114,115].

The antioxidant load in OMVs, including catalase (KatA), which is seven times more abundant on OMVs than in the outer membrane, is crucial to neutralize host-generated reactive oxygen species (ROS), particularly hydrogen peroxidase (H_2_O_2_), from neutrophils and polymorphonuclear leukocytes [116]. Meanwhile, OMVs can also cause oxidative stress by *Helicobacter pylori* neutrophil-activating protein (HP-NAP)-induced nicotinamide adenine dinucleotide phosphate (NADPH) oxidase activation, which produces a paradoxical pro-inflammatory milieu conducive to bacterial persistence [117,118].

Interestingly, OMVs cause the M2 polarization of macrophages, a tissue repair and immune tolerance-related phenotype through CD206 upregulation and CD86 downregulation, the increased production of IL-10 and IL-4, and the decreased production of IL-12 and IFN-γ [119,120]. All these reinforce an immunosuppressive microenvironment favorable for chronic infection. OMVs also modulate T cell survival through the induction of apoptosis in CD4^+^ and CD8^+^ subsets by cyclooxygenase-2 (COX-2) induction and prostaglandin E2 (PGE2) synthesis pathways suppressing T cell growth and IL-2 production [121,122]. VacA-enriched OMVs can also disrupt the immunological synapse and cause cytoskeletal degradation, nuclear factor of activated T-cells (NFAT) suppression, and glutamine starvation through gamma-glutamyltransferase (GGT), disrupting T cell function [123]. These vesicles also participate in extragastric diseases like liver fibrosis, cardiovascular disease, and neurodegeneration by modulating immune responses or transporting bacterial products to target tissues [124].

OMVs also play a significant role in *H. pylori* biofilm formation. For example, OMVs of the TK1402 isolate induce biofilm formation in a dose-dependent manner by acting as a structural backbone that provides orienting signals to bacterial cells and extracellular matrix components [49]. The coating of biofilm surfaces by OMV has been demonstrated using transmission electron microscopy, and proteomic profiling defined OMPs such as AlpB are required for adhesion and stability. Defective biofilm formation in AlpB mutants is corrected upon AlpB reintroduction [125]. Adhesin-enriched OMVs bearing adhesins such as BabA also promote early surface attachment, a critical event during the initiation of the biofilm [58].

Biofilm OMVs ensnare antibiotics like AMX and TET, forming diffusion barriers and protecting dormant bacterial populations [126]. Bacterial OMVs contain higher levels of extracellular DNA (eDNA) than planktonic OMVs and contribute to matrix stability and nuclease resistance degradation [127]. OMVs also facilitate horizontal gene transfer by delivering eDNA for natural transformation in biofilms, disseminating resistance and virulence genes [128]. Notably, EVs from infected host cells sometimes harbor hybrid bacterial host signatures, reflecting a complex intercellular cargo exchange [129]. Size heterogeneity in OMVs is also linked to functional heterogeneity, influencing adhesion, matrix support, and signaling functions in the biofilm microenvironment [129].

OMVs convey antibiotic resistance through a variety of mechanisms. They facilitate horizontal gene transfer by delivering resistance genes and enzymes like β-lactamases that degrade antibiotics [130]. OMVs also serve as decoys, trapping antibiotics before they reach their destination [131]. Certain OMVs cause active drug extrusion, such as OMV-mediated bismuth extrusion, which enables *H. pylori* to tolerate sublethal levels of bismuth [131,132]. Interestingly, OMVs selectively protect against clarithromycin and levofloxacin but not against amoxicillin or metronidazole [133], indicating their targeted role in modulating drug susceptibility.

Overall, *H. pylori* OMVs are multifunctional nanoscale vesicles that are attributed to orchestrating biofilm formation, immune evasion, and antibiotic resistance. Their roles in the modulation of infection niche, chronic colonization, and interaction with the host highlight their importance as potential therapeutic targets. An improved understanding of OMV biogenesis and the mechanism of action could lead to identifying OMV-disrupting compounds or vaccines for the eradication of *H. pylori* infections. Table 3 summarizes the various roles of *H. pylori* OMVs in immunomodulation, biofilm formation, and antibiotic resistance.

## 6. The Influence of *H. pylori* on Gut Microbiota

The human gastrointestinal (GI) tract contains a remarkably diverse and dense microbial community, with bacterial cell estimates in the colon ranging from 10^11^ to 10^12^ per milliliter, ranking it among the most densely inhabited environments on the planet [134,135]. The gut microbiome comprises more than 3.3 million genes, 150 times more genes than humans’ own genome, generating thousands of metabolites, greatly exceeding the human genome’s 23,000 genes [136]; the bacterial diversity analysis showed that about 1000 bacterial species live in our gut, and a majority of them belongs to the divisions of *Bacillota* (formerly *Firmicutes*), which includes genera like *Lactobacillus*, *Clostridium*, and *Faecalibacterium*, playing a role in short-chain fatty acids (SCFAs) production, energy metabolism, and gut health. *Bacteroidota* (formerly *Bacteroidetes*) [137], which includes *Bacteroides* and *Prevotella*, is involved in carbohydrate fermentation and SCFA production. *Actinomycetota* (formerly *Actinobacteria*), notably including *Bifidobacterium*, has beneficial effects on gut health, enhancing the mucosal barrier and inhibiting pathogens. *Pseudomonadota* (formerly *Proteobacteria*), including *Escherichia* and *Enterobacter*, is often associated with dysbiosis when in high abundance. *Verrucomicrobiota* (formerly *Verrucomicrobia*), with *Akkermansia muciniphila* as a key member, is linked to maintaining mucosal integrity and anti-inflammatory effects [138]. The human gut microbiota also includes a small but important component of fungal and yeast species, such as *Saccharomyces cerevisiae*, which can influence gut health and immune responses [139]. Additionally, archaea, like *Methanobrevibacter smithii*, are involved in methane production, contributing to the overall balance of the gut microbiota [140]. The virome, which consists of bacteriophages (viruses that infect bacteria) plays a significant role in regulating bacterial populations and maintaining the balance of the microbiota [141]. These diverse microbial entities, although smaller in number, collectively contribute to the complexity and functionality of the gut ecosystem, influencing digestion, immunity, and overall health. This has uncovered the microbiota’s variety and richness, which enhance its function as a “superorganism” with metabolic and immune roles, interacting symbiotically with the host [142].

*H. pylori* infection significantly alters the composition of the gut microbiota, leading to dysbiosis that may create an environment conducive to gastric atrophy and intestinal metaplasia [143]. *H. pylori* produce urease, which increases gastric pH by breaking down urea into ammonia. This altered pH can make the stomach less hospitable for acid-tolerant beneficial microbes, leading to colonization by opportunistic pathogens [144]. Early research using in situ hybridization and bacterial culturing found increased growth of *Lactobacillus acidophilus* in *H. pylori*-positive patients, indicating an imbalance in the gut microbiota, as these bacteria might proliferate in response to the changes in gut pH and the disruption caused by *H. pylori* [145], while a reduction in *Clostridia* and anaerobes was observed in comparison to *H. pylori*-negative subjects, which may impact processes such as fermentation and immune system modulation, potentially leading to gastrointestinal symptoms or an increased risk of other conditions like colorectal cancer [146]. Subsequent studies using fecal 16S rRNA analysis confirmed these findings and revealed that *H. pylori* infection leads to a decrease in microbial diversity, including an increased abundance of genera like *Haemophilus* and *Ralstonia*, while genera such as *Parasutterella* and *Pseudoflavonifractor* are found in lower levels [147]. The decreased levels of *Pseudoflavonifractor*, an SCFA producer, could negatively impact intestinal health, as SCFAs like butyrate are crucial for immune modulation and gut health [148]. Moreover, the infection is linked to a shift towards a *Prevotella*-dominated microbiome, regardless of dietary influences [149]. Other studies have reported that *H. pylori* infection could also enrich the gut microbiota with specific genera, such as *Succinivibrio*, *Coriobacteriaceae*, *Enterococcaceae*, and *Rikenellaceae* [150]. Notably, a large population study demonstrated that *H. pylori*-positive individuals exhibited a higher microbial diversity, along with increased abundance of genera such as *Actinomyces*, *Gemella*, *Streptococcus*, and *Haemophilus* [151]. However, in individuals with *H. pylori* colonization, the microbiota shifts towards an enrichment of *Spirochetes* and *Proteobacteria*. Carcinogenic metabolites, such as N-nitroso compounds produced by *Staphylococcus*, *Lactobacillus*, and *Escherichia coli* were observed, are implicated in cellular mechanisms that promote inflammation and tumor angiogenesis [152]. A study found that *H. pylori* infection increases the abundance of bacterial families like Succinivibrionaceae, Coriobacteriaceae, Enterococcaceae, and Rikenellaceae, as well as fungal species such as *Candida glabrata*. These microbial shifts may disrupt the intestinal mucosal barrier, potentially contributing to early-stage colorectal carcinoma [153]. In elderly individuals, reduced beneficial bacteria like butyrate-producing *Clostridium* clusters contribute to gastric carcinogenesis. Gastric cancer microbiota commonly includes strains of *Streptococcus*, *Lactobacillus*, *Veillonella*, and *Prevotella*, with specific *Streptococcus* species prevalent in gastric carcinoma [154]. Altered microbial communities in gastric cancer are linked to increased *Proteobacteria*, *Lactobacillus*, *Clostridium*, and *Rhodococcus*, highlighting the microbiota’s role in disease progression [155]. Persistent inflammation or atrophy in some patients was associated with shifts in microbial clusters, including increased *Acinetobacter* and *Streptococcus anginosus* [156]. Studies have also suggested that factors such as age, BMI, sex, and diet influence gut microbiota composition, highlighting the importance of controlling for these variables when evaluating the impact of *H. pylori* [157]. Overall, *H. pylori* infection alters gut microbiota diversity and composition, while eradication therapy involving broad-spectrum antibiotics significantly impacts microbial richness and specific populations. These shifts due to therapy affect metabolic health, microbial balance, and antibiotic resistance [158]. Although these microbial alterations are usually temporary, with the microbiota often returning to baseline within eight to ten weeks post-treatment, the recovery process can vary among individuals. The change in gut population includes an increased abundance of Enterobacteriaceae, Clostridiaceae, and certain Proteobacteria, alongside decreased levels of beneficial genera like *Bifidobacterium*, *Faecalibacterium*, *Actinobacteria*, and *Lactobacillus*, leading to temporary or prolonged dysbiosis, affecting gut barrier function and immunity [159]. Specifically, triple therapy moderately reduces diversity with notable increases in Enterobacteriaceae, whereas quadruple therapy causes broader microbial disruption. MTZ-containing regimens promote facultative anaerobes, and bismuth-based treatments are associated with prolonged dysbiosis, leading to B vitamins and folate, leading to deficiencies, and underscoring the variable impacts of different regimens [160]. *Megamonas* and the Rikenellaceae family, known for SCFA (butyrate and propionate) production, demonstrate alterations linked to improved glucose homeostasis after eradication therapy [161], while the use of antibiotics in eradication therapies has been associated with an increase in antibiotic resistance genes. For example, genes like *ermB* (macrolide resistance) and *tetQ* (TET resistance) have been identified; however, in some cases, the *tetO* and *tetW* were found to be decreased [162]. Eradication regimens also promote transient increases in genes like *ermB*, *CFX*, and *tetQ*, particularly after LVX-based therapies [163]. Another study found increases in opportunistic pathogens, such as *Enterococcus faecium* and *Clostridium difficile*, and a notable rise in antibiotic resistance genes [164]. This rise poses challenges for future treatment options and highlights the need for judicious antibiotic use. Table 4 provides the microbial shifts in response to *H. pylori* infection, indicating changes in bacterial and fungal genera and their implications on gut health, microbial diversity, and potential links to gastric and colorectal cancer.

## 7. Potential Treatment for Microbiota Recovery After *H. pylori* Infection

Current clinical practice guidelines recommend the use of combination regimens for the eradication of *H. pylori*, of which PPIs together with antibiotics such as clarithromycin, amoxicillin, or metronidazole form the basis of triple therapy. Quadruple therapy with bismuth and TET is applied in cases of antibiotic resistance or treatment failure [165]. The efficacy of these regimens is declining globally due to increasing antibiotic resistance, necessitating the pursuit of alternative or adjunct therapies [166]. Furthermore, conventional eradication therapies tend to induce drastic alterations in gut microbiota composition, resulting in dysbiosis, compromised gut barrier function, and increased susceptibility to secondary infection [167]. In response to these challenges, microbiota-sparing strategies have been proposed. Emerging evidence suggests that incorporating adjunctive therapies such as probiotics, prebiotics, or faecal microbiota transplantation (FMT) can accelerate the restoration of microbial balance and improve clinical outcomes [168,169].

Probiotics produce antimicrobial substances that directly inhibit the growth of *H. pylori*, such as acetic acid and lactic acid, H_2_O_2_, and bacteriocins. These products lower gastric pH, annihilate *H. pylori* cells, and suppress bacterial growth [170]. Probiotics, particularly *Lactobacillus* strains such as *Lactobacillus plantarum*, *Lactobacillus reuteri*, and *Lactobacillus casei*, also compete with *H. pylori* for adherence sites on the gastric mucosa and nutrients, suppressing colonization through the formation of protective barriers and the exclusion of pathogenic bacteria [171,172,173]. *Saccharomyces boulardii* is also particularly effective against the growth inhibition of *H. pylori* by modulating the immune response to *H. pylori*, restoring the gut microbiota balance disrupted by infection and antibiotics, and reducing the abundance of antibiotic-resistance genes [174]. In addition to their direct antimicrobial activity, certain *Lactobacillus* strains can stimulate the production of mucin, a protective coating in the stomach that prevents *H. pylori* from binding to gastric mucosa by modulating the immune response to better control infection [175,176]. A study found that *Lactobacillus plantarum* strains Q21, Q25, and QA85 were found to reduce *H. pylori* load, improve gastrointestinal symptoms, and modulate inflammation, but they cannot fully eradicate *H. pylori* when used as monotherapy [177]. Thus, the application of probiotics in conjunction with standard eradication therapies, such as triple therapy or PPIs, has been shown to improve eradication rates and reduce adverse effects [178]. Combinations like *Bifidobacterium–Lactobacillus* and *Bifidobacterium–Lactobacillus–Saccharomyces* have had the best overall performance in improving both eradication and side effect outcomes, whereas *Lactobacillus–Propionibacterium* was efficacious in eradication but with increased side effects [179]. Furthermore, multi-strain probiotic combinations, particularly those with *Saccharomyces boulardii* and combinations like *Clostridium butyricum* with *Bacillus coagulans*, have shown increased efficacy compared to single strains in the treatment of gastrointestinal disorders and *H. pylori* eradication [180]. The combinations have also shown promise with minimal side effects. Strain variability in efficacy and the need for further research into the dosing and duration of treatment are, nevertheless, still hindrances to their clinical application [181].

Autoprobiotics represent a novel approach to probiotic therapy, which are strains of indigenous microbiota recovered from the individual’s own body. The personalized strain formulation is tailored to the individual’s microbial milieu, which renders autoprobiotics more potent and capable of persisting in the gut for longer periods of time [182]. This reduces the requirement for prolonged treatments in comparison with conventional probiotics. Experimental evidence suggests that autoprobiotics, such as *Lactobacillus*, *Bifidobacteria*, or *Enterococci*, can accelerate the recovery of microbiota in dysbiosis induced by antibiotics in rats. In *H. pylori* chronic gastritis, autoprobiotics using native non-pathogenic *Enterococci* achieved 80% eradication and 100% symptom elimination in 20 days [183].

In addition, EVs derived from probiotics are also emerging as important mediators of bacteria–host interactions, offering potential therapeutic applications [184]. EVs carry bioactive molecules like proteins, lipids, and RNA that can shape immune responses, affect inflammation, and restore gut microbial homeostasis [185,186,187]. Further studies are needed to optimize their utilization in the clinic. EVs, particularly from *Lactobacillus crispatus* strains, are shown to modulate immune responses by regulating pro-inflammatory and anti-inflammatory cytokines [188]. For instance, EVs from *L. crispatus* significantly reduce pro-inflammatory cytokines IL-1β, IL-6, IL-8, and TNF-α while elevating the levels of anti-inflammatory cytokines IL-10 and TGF-β. Immunomodulation by this action averts excessive inflammatory reaction and tissue damage caused by *H. pylori* and, thereby, reduces gastritis and potentially improves healing in gastric epithelial cells [189]. Probiotic EVs are also involved in the maintenance of gastric and intestinal barrier function, an important determinant of gastrointestinal mucosa safety against pathogen invasion. EVs from *Lacticaseibacillus* species, such as *L. casei* and *L. rhamnosus*, contain proteins like p40 and p75 that prevent epithelial cell apoptosis and maintain tight junctions [190]. These proteins are responsible for keeping the mucosal surface intact and preventing pathogen-caused damage, thereby developing a protective barrier against *H. pylori* colonization [191]. Probiotic EVs can carry bacteriocins that target and inhibit pathogenic bacteria. While direct evidence of bacteriocins from probiotic EVs inhibiting *H. pylori* is still being explored, the antimicrobial properties of these molecules suggest a potential role in reducing bacterial load or preventing colonization [192]. The delivery of these antimicrobial agents via EVs enhances the targeted and sustained delivery to infected areas, such as the gastric mucosa, potentially improving therapeutic outcomes. Probiotic EVs also interact with immune cells, including macrophages. These interactions have the potential to reprogram macrophage polarization into an anti-inflammatory phenotype, enhancing their activity in phagocytosis and killing bacterial pathogens [193]. Such immunomodulation is particularly beneficial in controlling *H. pylori* infection and reducing the consequential tissue damage, further indicating the potential of probiotic EVs to control the infection and promote healing.

Prebiotics are non-digestible food ingredients that selectively stimulate the growth and activity of beneficial gut bacteria, particularly *Bifidobacterium* and *Lactobacillus* species [194] that can produce antimicrobial substances (bacteriocins, lactic acid) that inhibit *H. pylori* growth. These compounds, primarily oligosaccharides and non-starch polysaccharides, resist digestion in the small intestine and reach the large intestine intact, where they are fermented by beneficial microorganisms [195]. Common prebiotics include fructo-oligosaccharides (FOS), inulin, and galacto-oligosaccharides, which are found in various fruits and vegetables [194]. Prebiotics offer several advantages over probiotics, including lower cost, reduced risk, and easier incorporation into the diet [195]. They are also heat-resistant, allowing them to be used in baked goods and other processed foods [196]. By promoting the growth of beneficial bacteria, prebiotics can improve gut health, enhance resistance to pathogens, and confer various health benefits to the host [197]. Prebiotics stimulate beneficial gut bacteria, leading to increased SCFAs, such as butyrate, which lowers gastric pH and inhibits *H. pylori* growth. This effect is often associated with the increased population of beneficial bacteria like *Lactobacillus* and *Bifidobacterium*, which are stimulated by prebiotics [198], leading to the promotion of balanced gut microbiota. Prebiotics strengthen the mucosal barrier and reduce gastric inflammation [197]. While prebiotics alone are not sufficient enough to eradicate *H. pylori*, combining probiotics and prebiotics (synbiotics) improves gut microbiota and immune response, making it more effective than probiotics alone for supporting eradication therapy, but more robust studies are needed for definitive confirmation [199]. For an example, the combination of probiotics and prebiotics like butyric acid and inulin has also shown promise in reducing adverse events during *H. pylori* eradication treatment [197,198,199,200].

Similarly, FMT has shown promise in restoring microbiota composition and function by replenishing lost microbial diversity [201]. However, its role as a prophylactic measure to prevent bacterial infections in high-risk individuals remains under investigation. A pilot study on washed microbiota transplantation (WMT), another type of FMT for *H. pylori* eradication, showed a 40.6% eradication rate in patients. The study found that a higher pre-WMT PGR (probiotic growth ratio) was associated with a greater likelihood of successful eradication [202]. In a randomized controlled trial, patients receiving standard bismuth quadruple therapy (BQT) followed by a single FMT dose showed alleviation of gastrointestinal symptoms such as diarrhea compared to the placebo, although FMT did not significantly accelerate gut microbiota restoration. Post-antibiotic FMT can improve patient comfort and potentially reduce adverse effects associated with antibiotic regimens for *H. pylori* eradication [203]. Safety is a critical aspect of FMT, with potential risks including pathogen transmission from donors. Rigorous screening processes and large randomized controlled trials are requisite to confirm efficacy, refine protocols, and clarify mechanisms through which FMT cures *H. pylori* infection [204]. Adding FMT to conventional eradication therapy could enhance overall treatment success and counteract antibiotic-associated dysbiosis and resistance. Post-eradication management should not be overlooked; it should include non-invasive confirmatory testing (urea breath test or stool antigen test) to verify successful eradication, along with short-term microbiome restoration strategies such as targeted probiotic supplementation to mitigate lingering dysbiosis and reduce recurrence risk.

Probiotics, particularly *Lactobacillus*, *Bifidobacterium*, and *Saccharomyces boulardii*, have been extensively studied for adjunctive therapy in *H. pylori* eradication treatment. A meta-analysis of 45 randomized controlled trials in about 7000 patients showed that probiotic supplementation increased eradication rates by approximately 13% and significantly reduced common gastrointestinal side effects like diarrhea, nausea, and bloating (relative risk ~0.59) [205]. A further meta-analysis of networks encompassing 5792 patients reinforced these findings, confirming a statistically significant increase in eradication outcomes with the incorporation of probiotics. *S. boulardii*, in specific, has been shown to be quite effective in reducing treatment side effects and to modestly improve eradication when supplemented with routine triple therapy [206]. Preclinical studies further corroborate these findings because in vitro and animal models have proven that probiotic strains have efficacy in enhancing gut barrier function, influencing mucosal immunity, producing antimicrobial substances such as lactic acid and bacteriocins, and competing with *H. pylori* adhesion to gastric mucosa, which results in reduced bacterial burden and inflammation [207]. Autoprobiotics represent a new personalized treatment, with early clinical trials showing promising results. For instance, a pilot study of indigenous *Enterococcus faecium* achieved an 80% eradication rate and symptom improvement in *H. pylori*-associated chronic gastritis patients, although these results need to be confirmed by large-scale, controlled clinical trials [208]. Autoprobiotics, using animal models, have been found to have the ability to rapidly reconstitute the microbiota community following antibiotic-induced dysbiosis and confer anti-inflammatory activity. Another new approach is employing probiotic-derived EVs, but their clinical application is yet in its early stages. To date, no human clinical trials have assessed the efficacy of these EVs on *H. pylori* therapy. Preclinical evidence shows that EVs from species like *Lactobacillus crispatus* can modulate the inflammatory response, enhance the integrity of epithelial barriers by p40/p75 proteins, and regulate cytokine production, IL-1β, IL-6, IL-8, and TNF-α suppression, and stimulation of IL-10 and TGF-β, highlighting their immune-modulating nanocarrier properties. Prebiotics such as non-digestible fibers such as fructo-oligosaccharides (FOS), inulin, and galacto-oligosaccharides have limited clinical evidence on their own but are well established in preclinical studies for their ability to selectively stimulate the growth of beneficial microbes such as *Lactobacillus* and *Bifidobacterium*, enhance short-chain fatty acid production (butyrate), and promote mucosal and immune health [209]. Synbiotics, the combination of prebiotics and probiotics, are synergistically useful and have been shown to be clinically associated with reduced inflammation, enhanced mucosal immunity, and augmentation in numbers of health-promoting gut bacteria [210]. These are, however, formulation-dependent, and, more importantly against *H. pylori*, very few such trials have been conducted. FMT is established for recurrent *Clostridioides difficile* infection but remains under investigation in its application in the eradication of *H. pylori*. A pilot study using washed microbiota transplantation (WMT) reported a 40.6% rate of eradication and remission of symptoms but did not significantly accelerate the reconstitution of the microbiome compared to controls [211]. Preclinical models in animals have shown that FMT can reproducibly reconstitute microbial diversity and alleviate dysbiosis, holding promise for its future use as a microbiota-sparing intervention. All these microbiome therapies represent an intriguing future prospect for *H. pylori* management, with a progressively defined spectrum of clinical maturity, from well-established probiotics to new probiotic-derived EVs, and they emphasize the need for further randomized controlled trials to refine their roles and optimize incorporation into current treatment regimens. Table 5 shows the potential treatments for microbiota recovery after *H. pylori* infection.

## 8. Emerging Non-Antibiotic Therapies

Phage therapy has emerged as a promising alternative treatment for *H. pylori* infections, especially given the increasing antibiotic resistance of the bacterium, which affects over half of the global population and is associated with several gastric diseases [212]. The use of bacteriophages, particularly lytic phages, offers several advantages over traditional antibiotic treatments, including high specificity, safety, and the ability to rapidly adapt to bacterial resistance [213]. A study demonstrated that phages could resist simulated gastric juice when carried by *H. pylori*, making them a feasible treatment option and able to retain their ability to infect and destroy *H. pylori* cells [214]. Combining *H. pylori* phages with protective carriers like lactoferrin adsorbed on hydroxyapatite nanoparticles has been shown to enhance phage antibacterial activity and protect phages from gastric acidity [215]. Despite these promising findings, there is a lack of comprehensive phage collections, and phage resistance is a concern. Additionally, further research is needed to identify and characterize more lytic phages for use in therapy, as many studies have focused on the potential of prophages, which do not have the same therapeutic applications as bacteriophages of *H. pylori*.

Photodynamic therapy (PDT) has emerged as a promising alternative treatment for *H. pylori* infections, addressing the growing concern of antibiotic resistance, which utilizes the photosensitizing agents and light to generate reactive oxygen species that inactivate bacteria [216]. In vitro studies demonstrated that violet light (405 nm) alone was particularly effective in eradicating *H. pylori*, even without the use of added photosensitizers. This suggests that light-induced PDT can be a non-invasive, powerful treatment option [217]. Clinical trials have shown that PDT can reduce *H. pylori* load in the stomach, with the greatest effects observed in the antrum, the part of the stomach most commonly affected by *H. pylori* [218]. New developments, such as ingestible PDT capsules, have significantly improved the delivery of PDT to treat *H. pylori* infections, with some studies reporting up to 96% bacterial killing efficiency [219]. However, further research is required to optimize PDT protocols, refine safety, and establish its long-term efficacy as a mainstream treatment option for *H. pylori* eradication [220].

**Figure 3:**
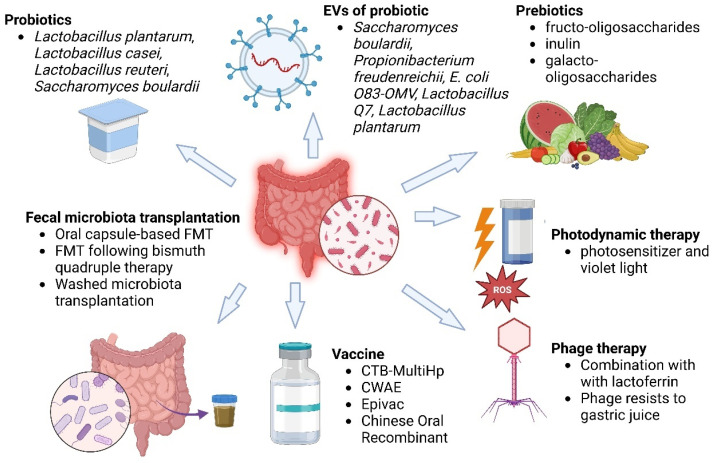
The various treatments against *H. pylori*. This figure shows some of the new strategies against *H. pylori* by microbiota modulation and adjunct therapies. The key strategies are listed as follows: Probiotics (*Lactobacillus plantarum*, *L. casei*, *L. reuteri*, *Saccharomyces boulardii*) are used to inhibit *H. pylori* colonization and gut health. EVs of probiotics derived from strains like *S. boulardii*, Propionibacterium freudenreichii, and *L. plantarum* can have antimicrobial and immunomodulatory activities. Prebiotics (fructo-oligosaccharides, inulin, galacto-oligosaccharides) enhance the growth of favourable flora. FMT via oral capsules or post-bismuth treatment restores gut microbial homeostasis. Vaccines (CTB-MultiHp, CWAE, Epivac, Chinese oral recombinant vaccines) are under development for *H. pylori* prevention. Photodynamic treatment with a photosensitizer and violet light can generate ROS and eliminate *H. pylori*. Phage therapy, often in combination with lactoferrin, can be used for *H. pylori* with phages that are resistant to gastric acidity. All these strategies reflect a multidisciplinary approach to combat antibiotic resistance and promote effective *H. pylori* eradication.

Despite decades of research, a widely available vaccine for *H. pylori* remains elusive, although significant progress has been made. The most impressive achievement was the development of an oral recombinant vaccine in China, which was approximately 72% effective against natural *H. pylori* infection in children in a Phase 3 clinical trial [221]. The vaccine employed the urease subunit B (UreB) with the heat-labile enterotoxin B subunit (LTB) as a mucosal adjuvant. Despite being successful, its development was ceased, showing the complex path from clinical usefulness to commercial success [222]. Many other contenders have emerged in preclinical phases. Multi-antigenic and multi-epitope vaccines such as CTB-multiHp have a strong immune reactivity and are being considered further for their protective function [223]. A whole-cell *H. pylori* inactivated vaccine combined with a non-toxic mucosal adjuvant (mmCT) significantly reduced bacterial colonization in mouse models [224]. Similarly, a multivalent epitope-based vaccine (CWAE) targeting heterogeneous *H. pylori* antigens exhibited therapeutic effects in Mongolian gerbils via the inhibition of colonization and robust immune responses [225]. Another promising candidate, Epivac, a protein CD4+ T cell epitope-based vaccine containing *H. pylori* protein, induced Th1-skewed immune responses and provided protection in mice [226]. In addition to classic antigens such as urease, VacA, and CagA, newer strategies are examining immune evasion targets such as γ-glutamyltranspeptidase (GGT) to make vaccines more effective. Advances in immunoinformatics and molecular docking have enabled the design of non-allergenic, stable multi-epitope constructs that can stimulate immune receptors like Toll-like receptors (TLRs) 2 and 4 [227]. The majority of these candidates, though, are effective in animal models but pose a difficult task to translate preclinical success into an effective human vaccine. The absence of natural sterilizing immunity, early age of acquisition, and *H. pylori*’s strategies for evading the host’s immune response all add to the challenge. However, with novel adjuvants, nucleic acid platforms (DNA and mRNA vaccines), and artificial intelligence-informed design tools, it is now more feasible to construct a safe, effective, and universally accessible *H. pylori* vaccine. According to experts’ estimates, the vaccine will be available within 8–10 years, only if research is funded regularly and it is kept on the top of the world’s health agenda. Ongoing research focuses on identifying protective immune mechanisms and optimizing vaccine formulations for broad protection. Figure 3 shows the overall available and potential treatments to treat the infection.

## 9. Natural Products as Promising Alternative Agents for *H. pylori* Eradication

Natural products have emerged as a promising multi-targeted strategy to combat *H. pylori*, especially in the context of rising antibiotic resistance, biofilm-associated persistence, and virulence-driven inflammation. By targeting many pathways, several phytochemicals including phenols, terpenoids, flavonoids, alkaloids, and bee-derived molecules have shown strong anti-*H. pylori* action [228,229,230].

Particularly, biofilms shield *H. pylori* from host immune clearance and antibiotics, and natural products have demonstrated significant potential in disrupting such structures. Several natural molecules have shown promising antibiofilm activity through different mechanisms. Eugenol, a phenolic component of clove oil, destabilizes bacterial membranes, quenches quorum sensing, and downregulates key virulence genes such as *cagA* and *vacA* [228]. Terpenoids thymol and carvacrol, present in *Thymus kotschyanus* and *Origanum vulgare*, respectively, destabilize membranes, inhibit ATP synthesis, and inhibit biofilm maturation [229]. Myricetin and curcumin are polyphenols with potent antibiofilm activities that compromise urease activity, enhance immune recognition, and modulate NF-κB and MAPK inflammatory pathways [230]. Cumin, rosemary, cinnamon, and ylang-ylang essential oils (EQs) are a good number of oils from plants that interfere with *H. pylori* biofilms by disrupting adhesion and matrix stability [231]. Ginger extract at low concentrations inhibits biofilm formation by over 90% and possesses antibacterial and anti-inflammatory activities [231]. *Aloe-emodin* targets outer membrane proteins that are essential for the architecture of biofilms, induces oxidative stress, and destabilizes established biofilms [232]. Green tea coumarins (*Camellia sinensis*) suppress bacterial adhesion, DNA gyrase, and quorum sensing signals [233]. These compounds target by multiple mechanisms that encompass membrane disruption, quorum sensing inhibition, urease inhibition, silencing virulence genes, and immunomodulation, with the additional advantage of synergizing with conventional antibiotics [234,235]. Together, natural products represent a multimodal approach to the therapy of *H. pylori* infection, including biofilm-related resistance, and a compelling platform for adjunctive or alternative treatment.

Natural products also act as inhibitors of urease for *H. pylori*. The urease enzyme is central to the pathogenicity and survival of *H. pylori* by hydrolyzing urea into ammonia and carbon dioxide, which in turn neutralizes stomach acid and supports the colonization of the acidic stomach lining [144]. Urease is also involved in mucosal inflammation, immune evasion, and epithelial injury. The inhibition of urease is hence a powerful strategy against *H. pylori* infection, that is, one which is specific to antibiotic resistance [236]. Natural compounds with diverse structures, for example, flavonoids, polyphenols, coumarins, and phytochemicals, have been found to possess remarkable urease inhibitory activity with diverse mechanisms. The most promising inhibitors of urease are flavonoids and polyphenols, which are well known to exhibit antioxidative and antimicrobial activity, and they also possess in vitro urease inhibitory effects, with IC_50_ values ranging from approximately 11 to 100 µM [237]. Genistein, an isoflavone, a member of the Fabaceae plant, exhibits the 50% inhibition of urease at 430 µg/mL, whereas baicalin and scutellarin, flavone glucuronides of *Scutellaria baicalensis* and *Erigeron breviscapus*, have been found to chelate directly to the active sites of the enzyme [238]. Particularly notable are methyl gallate and penta-O-galloyl-β-D-glucoside (PGG) from *Paeonia lactiflora* roots, whose activity is almost as effective as the established synthetic urease inhibitor acetohydroxamic acid [239]. Certain plant extracts have also been examined for anti-urease activity. Methanolic extracts of *Camellia sinensis* (green tea), rich in epigallocatechin gallate (EGCG) and other related catechins, have very high urease inhibitory activity in vitro, with an IC_50_ of around 13 µM [240]. Other phenolic-rich plant extracts of *Punica granatum* (pomegranate), *Origanum vulgare* (oregano), *Vaccinium macrocarpon* (cranberry), *Matricaria recutita* (chamomile), and *Nasturtium officinale* (watercress) have also exhibited significant urease inhibition, largely as a result of their presence of flavonoids and tannins [241]. *Ginkgo biloba* extract showed very good efficacy with an IC_50_ of 36.17 µg/mL, which was better than the positive control hydroxyurea and *Rhus coriaria* (sumac) extract inhibited at 80.29 µg/mL, consistent with its ancient antimicrobial use in gastrointestinal disease [242]. Zerumbone, a sesquiterpene derived from *Zingiber zerumbet* (wild ginger), has also inhibited *H. pylori* urease activity effectively, but its precise IC_50_ is not given [243]. Mechanistically, naturally occurring urease inhibitors function primarily by binding the nickel-containing catalytic site of the enzyme or with sulfhydryl (-SH) groups of cysteine residues, obstructing substrate access and enzymatic activity. Flavonoids and polyphenols predominantly are metal-chelating agents that sequester the essential metal ions in the active site. Other compounds suppress urease gene expression or disrupt the quaternary structure of the enzyme, contributing to its dysfunction. Through this, these agents debilitate the ability of the bacterium to resist gastric acid, reduce colonization competence, and enhance vulnerability to immune clearance and antibiotics [244]. The large diversity of natural compounds with urease inhibitory activity emphasizes their therapeutic potential. They can also be applied as adjuvants to conventional triple or quadruple therapy, potentially lowering the doses of antibiotics needed and reducing resistance [245]. Moreover, their immunomodulatory as well as antioxidant activities may help in mucosal healing and resolving inflammation.

In terms of membrane-targeting activity, natural compounds such as coptisine (from *Rhizoma coptidis*) compromise *H. pylori* viability by exteriorizing phosphatidylserine, inducing DNA fragmentation, and damaging virulence factor expression [246]. Similarly, sanguinarine (from *Zanthoxylum nitidum*) causes cell lysis and urease inhibition, while squalamine, a steroidal alkaloid, compromises membrane integrity and increases antibiotic permeability [247]. Piperine interferes with motility and adhesion, impairing the colonizing capacity of the bacterium. Terpenoid-containing EQs such as thymol, carvacrol, and eugenol interact with lipid bilayers, permeabilize, and interfere with membrane-bound processes such as efflux and virulence [248]. Phytochemicals such as kaempferol, tellimagrandin I, curcumin, and propolis-derived flavonoids also destabilize membrane integrity. *Aloe-emodin* and ginger phenolics also play a role in membrane dysfunction, proving to be valuable adjuvants in the eradication of drug-resistant *H. pylori* strains [249].

Several plant extracts and purified molecules have been found to demonstrate quorum-sensing inhibitory activity against *H. pylori*. MeMe of *Chelidonium majus* and *Corydalis cheilanthifolia* has shown significant in vitro QS inhibition against a number of *H. pylori* strains [250]. Likewise, *Atractylodes lancea* volatile oils and *Pistacia vera oleoresins* have shown in vitro and in vivo anti-QS activity and are thus potential systemic anti-virulence agents [251]. In silico docking studies have identified a series of bioactive phytochemicals that include β-carotene, β-amyrin, taraxasterol, bauerenol, taraxacin, and benzoyl peroxide as highly potent binders to the *H. pylori* chemoreceptor TlpB, the central sensor of the AI-2-mediated QS system. The binding of these molecules to TlpB effectively inhibits the perception of AI-2, which in turn affects signal transduction, gene regulation, and ensuing pathogenic processes [252,253]. These compounds also bear promising pharmacological attributes like low toxicity and good bioavailability in model systems. EQs and their major constituents are also another vital category of natural QS inhibitors. The EOs of essential herbs of the Lamiaceae family, including *Origanum vulgare* (oregano), *Thymus vulgaris* (thyme), and *Rosmarinus officinalis* (rosemary), have exhibited significant anti-QS and antibiofilm activities against *H. pylori* and related pathogens [254]. Key EO constituents such as carvacrol, thymol, eugenol, linalool, limonene, γ-terpinene, and 1,8-cineole disrupt QS-regulated behavior by inhibiting AI-2 synthesis, transport, or binding to receptors. Lipophilic molecules such as these can diffuse through bacterial membranes, interfere with signaling proteins, and rewire gene expression patterns governing adhesion, motility, and toxin production [255].

Flavonoids and furanones have also been found to have strong QS inhibitory activity. While certain flavonoids such as *H. pylori* QS are likewise yet to be well understood, their broad-spectrum QS inhibition in other models of bacteria suggests similar modes of action. These compounds typically act by competing with AI-2 at the receptor level, blocking signal perception, or inhibiting LuxS-dependent AI-2 biosynthesis [256]. Synthetic furanones that mimicked natural halogenated furanones of marine algae were well documented to inhibit QS in Gram-negative bacteria by facilitating the degradation of signal receptors or the simulation of signal molecules to competitively inhibit binding [257].

The inhibition of virulence gene expression in *H. pylori* is an excellent method used to combat its pathogenicity, limit tissue damage, and increase eradication rates. Virulence factors such as CagA, VacA, urease, outer membrane adhesins, and biofilm-related genes play a key role in *H. pylori*’s ability to colonize the gastric mucosa, evade immune detection, and induce chronic inflammation. A wide range of natural products, particularly alkaloids, polyphenols, flavonoids, terpenoids, and bee-derived compounds, have demonstrated the capacity to suppress these virulence-related genes and pathways through multiple mechanisms [258]. Eugenol not only exhibits strong antibiofilm and membrane-disruptive properties but also downregulates the expression of key virulence genes, attenuating the ability of *H. pylori* to establish persistent infection [259]. Similarly, terpenoids such as thymol and carvacrol, respectively, interfere with membrane integrity and pH homeostasis, thus indirectly inhibiting the expression of virulence genes related to biofilm and adhesion. Alkaloids such as coptisin (from *Rhizoma coptidis*) have also been found to suppress the expression of the Cag pathogenicity island. Sanguinarine, which is another *Zanthoxylum nitidum* alkaloid, exhibits urease inhibition and cell lysis, contributing to diminished virulence expression and bacterial burden [260]. Among the most active polyphenols and flavonoids, kaempferol inhibits bacterial membrane function, which inhibits virulence factor functionality. Chalcone derivatives, from plant families like Leguminosae, Asteraceae, and Moraceae, inhibit *H. pylori* motility, adhesion, and urease activity, thereby downregulating virulence at structural and enzymatic levels [261]. Myricetin, from Myrica species, improves the immune recognition of *H. pylori*, sensitizes to antibiotics, and reduces biofilm formation, all of which are linked with suppressed virulence gene expression. Curcumin is also well known for urease inhibition and NF-κB inhibition, diminishing inflammation and indirectly suppressing the synthesis of virulence factors. Apigenin, a flavonoid in celery and parsley, also disrupts NF-κB pathways, thereby inhibiting the inflammatory damage caused by *H. pylori* virulence action [262]. Another important class includes bee-derived products, specifically propolis, which is rich in polyphenolic compounds like caffeic acid phenethyl ester (CAPE). CAPE has been shown to inhibit the bacterial peptide deformylase enzyme required for protein maturation, thus impacting *H. pylori* protein expression, including virulence-related proteins [263]. Moreover, extracts of *Acacia nilotica*, *Calotropis procera*, and *Geranium wilfordii* plants have been reported to display anti-urease and anti-adhesive activity, with evidence pointing towards their potential wider role of modulating virulence-associated gene networks [264]. In summary, natural products exert strong anti-*H. pylori* properties via a constellation of mechanisms, including biofilm disruption, urease inhibition, membrane weakening, virulence gene silencing, and quorum sensing blocking. Their multitarget activity, low resistance potential, and synergy with antibiotics underscore their value as adjunctive or alternative therapeutics. This integrated phytochemical approach, supported by both experimental research and mechanistic insight, paves the way for new strategies to cure *H. pylori* infection, especially those complicated by resistance or recurrence.

## 10. Conclusions and Future Perspective

**Figure 4:**
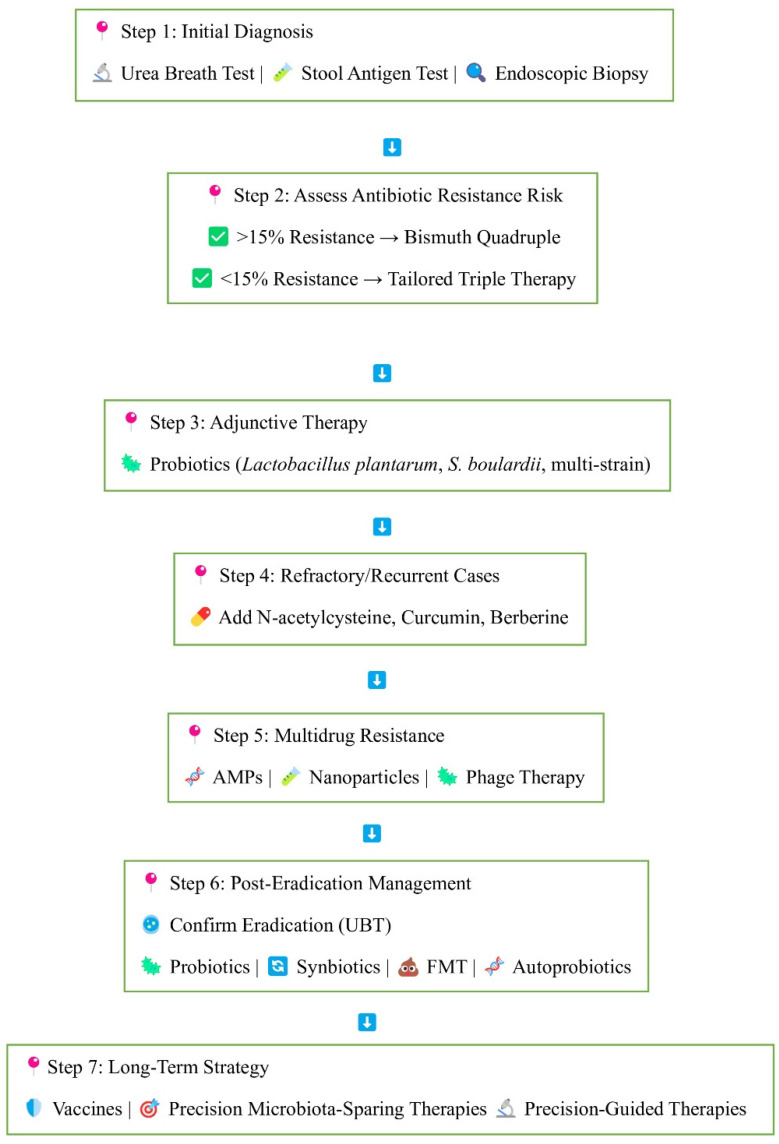
Stepwise approach to *H. pylori* eradication and microbiota recovery. The flowchart illustrates a comprehensive approach to *H. pylori* management. Step 1: Initial non-invasive (urea breath test, stool antigen test) or invasive (endoscopic biopsy) diagnosis. Step 2: Treatment is selected on the basis of local antibiotic resistance prevalence: Bismuth quadruple therapy for resistance >15%, tailored triple therapy for resistance <15%. Step 3: Probiotic adjunctive therapy (*Lactobacillus plantarum*, *S. boulardii*) may enhance efficacy and reduce side effects. Step 4: For treatment-resistant or recurrent infection, additional agents such as N-acetylcysteine, curcumin, and berberine are used. Step 5: For multidrug resistance, newer strategies include antimicrobial peptides (AMPs), nano-particles, and phage therapy. Step 6: Post-eradication confirmation by UBT is important, followed by the restoration of gut microbiota by probiotics, synbiotics, fecal microbiota transplantation (FMT), or autoprobiotics. Step 7: Long-term strategies focus on preventive and precision strategies such as vaccines, micro-biota-sparing therapy, and precision-guided therapies.

The successful eradication of *H. pylori* in the era of rising antimicrobial resistance requires a multifaceted, stepwise strategy. Clinicians should first consider region-specific antibiotic resistance patterns, prioritizing bismuth quadruple therapy in high clarithromycin resistance settings (>15%) and using tailored triple therapy where resistance remains low. The adjunctive use of probiotics, particularly strains like *Lactobacillus plantarum*, *Saccharomyces boulardii*, or multi-strain formulations, can enhance eradication rates and reduce gastrointestinal side effects, thereby improving adherence. In refractory or recurrent infections, combining antibiotics with biofilm-disrupting agents such as N-acetylcysteine or natural compounds (curcumin, berberine) may help overcome biofilm-associated persistence. For multidrug-resistant cases, emerging alternatives like AMPs, nanoparticle-mediated drug delivery systems, or phage therapy offer promising avenues, although further clinical validation is required. Long-term success will depend on the development of vaccines and microbiota-sparing interventions such as autoprobiotics, probiotic-derived EVs, and FMT to support microbiome recovery and reduce antibiotic-induced side effects. This also warrants further exploration through rigorously designed randomized trials. Furthermore, real-world clinical data reinforce the utility of microbiota-targeted adjuncts. For example, probiotic–antibiotic co-administration has been shown in multiple trials to reduce adverse events such as diarrhea, nausea, and bloating by 30–40%, thereby improving patient tolerance and adherence. Pretreatment resistance testing, especially in high-burden regions, remains crucial for guiding optimal therapy selection. Patient education should also be emphasized to improve compliance with complex multidrug regimens. In resource-limited settings, low-cost adjuncts such as standardized garlic or ginger extracts may serve as practical and accessible options to support eradication efforts. Lastly, integrating multi-omics approaches (metagenomics, transcriptomics, proteomics) may help to identify predictive biomarkers of treatment response and enable precision-guided interventions tailored to the individual patient’s microbiota and immune landscape. In addition, nanoparticle-based drug delivery systems and vaccines targeting *H. pylori* antigens hold even more promise in the fight against this devious pathogen. The future of *H. pylori* therapy is a part of an interdisciplinary approach involving alternative therapies, precision medicine, and enhanced antibiotic stewardship in addressing the challenge of resistance. Research into the role of microbiota in the dynamics of infection and the optimization of non-antibiotic treatment will be vital in overcoming the limitations of current treatment modalities. Finally, a more complete and individualized therapy regimen will be essential in controlling *H. pylori* infections and in diminishing the threat of gastric cancers and other such illnesses. As the resistance to antibiotics continues to climb worldwide, novel and adjunct treatments will increasingly be needed. Developing personalized, microbiota-tolerant, and sustainable treatment choices will be the key to the future of *H. pylori* treatment and worldwide efforts to counter antibiotic resistance. Figure 4 explains a stepwise algorithm integrating diagnosis, resistance-guided treatment, adjunctive support, and long-term microbiome recovery.

## Figures and Tables

**Table 1:** The global resistance patterns, mechanisms, and regional variations of antibiotics used against *H. pylori*.

Antibiotic	Global Resistance Patterns	Primary Resistance Mechanisms	Notable Regional Variations
Amoxicillin (AMX)	Generally low (0–10%)	PBP1A mutations (e.g., S402G, T555S); β-Lactamase production (blaTEM-1); Porin/efflux pump modifications (HopB, HefA, omp25).	- Asia: ~3%; - China: 2.8%; - Europe: ~0.4%; - Vietnam: 25.7%; - Africa: up to 100%.
Metronidazole (MTZ)	Moderate to high	Mutations in rdxA, frxA, fdxB genes; Reduced nitroreductase activity; Efflux pump genes (hp1165, hefA).	- China: 87.8%; - Bangladesh: 94.6%; - India: 81.6%; - Europe: 32% avg; - France: 58.6%; - Austria: 10.2%; - USA: 23%.
Clarithromycin (CLR)	Variable	23S rRNA mutations (A2142G, A2143G); RND family efflux pumps; OMP alterations (HopT, OMP31).	- Europe: 18%; - Eastern Mediterranean: 33%; - Western Pacific: 34%; - Americas: <10%; - Southeast Asia: <10%; - Africa: 29.2%; - Congo: 1.7%.
Tetracycline (TET)	Generally low	16S rRNA mutations; Proton motive force-dependent efflux pumps; Hp1165 gene expression.	- Global: 0.6–8%; - Europe: <1%; - Notable increases in Iran and China.
Levofloxacin (LVX)	Moderate to high	QRDR mutations in gyrA (N87K, D91G); gyrB gene mutations.	- China: 30.29%; - India: 54.9%; - USA: 37.6%; - Iran: 18%; - Lower rates in Pakistan, Thailand, Brazil.

**Table 2:** The differences in bEVs from Gram-negative versus Gram-positive bacteria.

Aspect	Gram-Negative Bacteria (e.g., *H. pylori*)	Gram-Positive Bacteria
Vesicle Type	Outer membrane vesicles (OMVs)	Cytoplasmic membrane vesicles (CMVs)
Membrane Structure	Outer membrane, thin peptidoglycan layer, and inner membrane	Cytoplasmic membrane with thick peptidoglycan layer
Vesicle Formation Mechanism	Blebbing (focal curvature and protrusion of the outer membrane leading to vesicle pinching off)	Bubbling cell death (bulging of cytoplasmic membrane and vesicle release via autolysins)
Cargo	LPS, phospholipids, proteins, toxins, and nucleic acids	Membrane-bound proteins, enzymes, toxins, PG fragments, and signalling molecules
Vesicle Function	Immune modulation, biofilm formation, horizontal gene transfer, nutrient acquisition, and stress response	Immune modulation, virulence factor transfer, biofilm formation, and communication with host cells
Role in Biofilm Formation	Critical for biofilm formation by promoting adherence and stabilizing bacterial colonies	Contributes to biofilm enhancement, particularly in the release of virulence factors that aid in biofilm stability
Bacterial Pathogenesis	OMVs play a key role in virulence by transporting virulence factors, immune modulators, and toxins that influence host immune responses and infection	CMVs contribute to bacterial pathogenesis through the transfer of toxins, enzymes, and immune evasion strategies
Vesicle Contents in Pathogenesis	Contains PAMPs (e.g., LPS) that stimulate immune responses and modulate host–pathogen interactions	CMVs transport a variety of virulence factors, including toxins and autolysins that promote infection and host modulation
Formation Under Stress	OMVs are formed as a stress response to environmental challenges such as nutrient limitation, oxidative stress, and host immune responses	CMVs are produced in response to stress or programmed cell death, facilitating intercellular communication or immune evasion
Biogenesis Regulation	Regulated by environmental cues, bacterial growth phase, and gene control	Regulated by environmental cues, stress conditions, and cell death mechanisms

**Table 3:** The key immunomodulatory, biofilm-related, and antibiotic resistance functions of *H. pylori* OMVs.

Functional Role	OMV Components/Mechanism	Impact	References
Immunomodulation	CagA, VacA, LPS, peptidoglycan	Activates PRRs (TLRs, NOD1) NF-κB signalling, cytokine release (IL-8, IL-6, TNF-α)	[110,111]
	HO-1 induction via Akt-Nrf2, mTOR–IKK–NF-κB pathways	Promotes anti-inflammatory macrophage phenotype (M2 polarization)	[112,119,120]
	VacA, GGT, snRNAs, nucleic acids	Induces T cell apoptosis, autophagy inhibition, antigen presentation disruption	[113,114,115,121,122,123]
	KatA, HP-NAP	Balances oxidative stress (detoxifies ROS; paradoxical inflammation via NADPH oxidase)	[116,117,118]
	Extragastric dissemination of OMV cargo	Implicated in liver, cardiovascular, neurodegenerative diseases	[124]
Biofilm Formation	Structural proteins (e.g., AlpB), adhesins (e.g., BabA), high eDNA levels	Promotes adhesion, ECM integrity, antibiotic shielding	[49,58,125,126,127]
	OMVs in ECM	Scaffold for 3D biofilm structure, protects against gastric acid and stress	[49,126]
	OMVs as quorum-sensing modulators	Regulates microcolony formation, biofilm dispersal	[49,127]
Antibiotic Resistance	β-lactamases, DNA fragments, membrane-bound enzymes	Enzymatic degradation of antibiotics (e.g., β-lactam antibiotics)	[130]
	OMVs as decoys	Sequester antibiotics, reduce their bioavailability	[131]
	OMV-mediated extrusion (e.g., bismuth)	Expels antimicrobials, facilitates tolerance to sublethal drug levels	[132]
	Selective shielding	Protects against clarithromycin, levofloxacin; not effective against amoxicillin, metronidazole	[133]

**Table 4:** Microbial changes induced by *H. pylori* infection.

Microorganism	Change	Implications/Outcome
*Lactobacillus acidophilus*	↑	Imbalance in gut microbiota, potentially proliferating in response to altered gastric pH and disruption.
*Haemophilus*	↑	Increased microbial diversity, potentially associated with inflammation and dysbiosis.
*Ralstonia*	↑	Indicative of microbial dysbiosis, potentially linked to gut inflammation and altered gut homeostasis.
*Succinivibrio*	↑	Enrichment linked to changes in microbial community composition and possible impact on intestinal health.
*Coriobacteriaceae*	↑	Associated with microbial shifts and potential contribution to gut inflammation and colorectal cancer.
*Enterococcaceae*	↑	Increase linked to microbial dysbiosis and possible impact on gut health and cancer risk.
*Rikenellaceae*	↑	Potential association with early stage colorectal carcinoma and altered gut microbial balance.
*Actinomyces*	↑	Enrichment potentially linked to microbial dysbiosis and related to gastric or colorectal health.
*Gemella*	↑	Microbial shift indicating dysbiosis, potentially influencing gastrointestinal inflammation.
*Streptococcus*	↑	Increased abundance, linked to gastric carcinogenesis and possibly promoting inflammation in the gastric mucosa.
*Spirochetes*	↑	Enrichment may suggest a shift towards a pathogenic microbiome associated with cancer risk and inflammation.
*Proteobacteria*	↑	Linked to microbial dysbiosis and inflammation, with a potential role in carcinogenic processes.
*Candida glabrata*	↑	Fungal species increase linked to disruption of the intestinal mucosal barrier and colorectal carcinoma.
*Clostridia*	↓	Reduced abundance of butyrate-producing bacteria, impairing gut health and immune modulation.
*Anaerobes*	↓	Decreased levels associated with disruption in microbial fermentation processes, impacting gut health.
*Parasutterella*	↓	Reduction could impact intestinal health, immune regulation, and microbial diversity.
*Pseudoflavonifractor*	↓	Reduction of SCFA-producing bacteria, potentially impairing gut health and immune system function.
Butyrate-producing bacteria	↓	Decrease in beneficial SCFA producers like *Clostridia*, impairing gut health and immune modulation.

**Table 5:** Summary of microbiota recovery strategies after *H. pylori* infection.

Therapy	Clinical Evidence	Preclinical Evidence	Mechanism of Action	Limitations/Considerations
Probiotics	Well-supported by meta-analyses; improves eradication rates (~13%) and reduces side effects	Enhances mucosal immunity, inhibits *H. pylori* adhesion, promotes antimicrobial activity	Produces SCFAs, bacteriocins, competes with *H. pylori*, strengthens epithelial barrier	Strain-specific efficacy; inconsistent results; optimal dosage/duration still under study
Autoprobiotics	Promising small-scale trials; 80% eradication and symptom relief with Enterococci	Accelerated microbiota restoration, reduced inflammation	Personalized strains colonize efficiently, enhance host compatibility	Limited clinical validation; requires personalized production and microbiota screening
Probiotic-Derived EVs	No current human trials	EVs modulate immune responses, cytokine balance, and barrier function	Deliver immunomodulators (e.g., p40/p75), cytokine modulation, suppress inflammation	Still experimental; scalable isolation and delivery systems need development
Prebiotics	Limited but emerging data in combination with probiotics	Selective stimulation of *Lactobacillus*, *Bifidobacterium*; SCFA production	Increases butyrate and lactate, improves mucosal immunity	Less effective alone; effect highly dependent on individual microbiota and diet
Synbiotics (Probiotic + Prebiotic)	Some clinical evidence supports reduced inflammation and improved immunity	Demonstrated synergistic effects on gut microbiota and immune regulation	Combine benefits of both probiotics and prebiotics	Formulation-dependent results; limited studies in *H. pylori*-specific settings
Fecal Microbiota Transplantation (FMT)	Pilot studies report ~40.6% eradication when used with standard therapy; improves GI symptoms	Resets microbial diversity; reverses dysbiosis	Replaces depleted microbiota, promotes ecological resilience	Safety concerns (pathogen transfer); requires standardization, donor screening, and further validation
